# Assessment of Physical Fitness after Bariatric Surgery and Its Association with Protein Intake and Type of Cholecalciferol Supplementation

**DOI:** 10.3390/medicina55060281

**Published:** 2019-06-17

**Authors:** Hendrika J. M. Smelt, Sjaak Pouwels, Alper Celik, Adarsh Gupta, Johannes F. Smulders

**Affiliations:** 1 Department of Surgery, Catharina Hospital, 5623 EJ Eindhoven, The Netherlands; marieke.smelt@catharinaziekenhuis.nl (H.J.M.S.); frans.smulders@catharinaziekenhuis.nl (J.F.S.); 2 Obesity Center, Catharina Hospital, 5623 EJ Eindhoven, The Netherlands; 3 Department of Surgery, Haaglanden Medical Center, 2512 VA The Hague, The Netherlands; 4 Metabolic Surgery Clinic, Sisli, 34340 Istanbul, Turkey; doktoralper@hotmail.com; 5 Center for Medical Weight Loss & Metabolic Control, Rowan University, Stratford, NJ 08084, USA; guptaad@rowan.edu

**Keywords:** physical fitness, shuttle walk run test, handgrip strength, vitamin D

## Abstract

*Background and objectives:* Several studies showed that there is a relationship between vitamin and mineral status and muscle strength. In particular this is the case for handgrip strength (HS) and vitamin D deficiency. In bariatric surgery there is a risk of decrease in muscle strength after surgery and also vitamin and mineral deficiencies are not uncommon. The aim of this study is to assess the effect of low vitamin 25 (OH) cholecalciferol levels, high dose cholecalciferol supplementation regime and protein intake on physical fitness, measured using handgrip strength (HS) and the shuttle walk run test (SWRT). *Materials and Methods:* For this retrospective study, 100 patients who have had bariatric surgery were included. Group A (n = 50) used 800 IU oral cholecalciferol per day. Group B (n = 50) used 800 IU oral cholecalciferol daily and 50,000 IU liquid cholecalciferol monthly lifelong. Both groups were matched on common variables. To measure physical fitness, we used the HS manometer of Jamar and the Shuttle Walk Run Test (SWRT) to assess physical capacity. *Results:* No significant differences in HS and SWRT outcomes were found between patients with serum 25 (OH) cholecalciferol < 75 nmol/L or >75 nmol/L. The postoperative HS is significantly influenced by protein intake (*p* = 0.017) and no significant influence was seen in outcomes of the SWRT (*p* = 0.447). *Conclusion:* We have found that serum 25 (OH) cholecalciferol and different cholecalciferol supplementation regimes do not have a significant effect on HS and SWRT before, three and 6 months after surgery. It seems that protein intake plays a more important role in maintaining adequate muscle strength.

## 1. Introduction

Obesity and associated comorbidities are growing worldwide to epidemic proportions and bariatric surgery is the only established treatment that provides a rapid and long-lasting weight reduction and a significant decrease in mortality and morbidity [1]. Some of the available literature suggests that bariatric surgery may also induce bone loss, despite adequate supplementation of vitamins and minerals. Some of the ‘post-bariatric’ patients may develop osteopenia, osteomalacia and osteoporosis [2]. In states of malnutrition, impaired muscle strength can also occur. Because of a low nutritional intake (especially proteins) after bariatric surgery, this may lead to a compensatory loss of (muscle) protein that is preferably lost from muscle mass, the body’s largest protein reserve [3]. Consequently, a reduction in muscle strength is associated with loss of physical capacity and function and also has a negative impact of recovery, especially after surgery. This explains the predictive aspects of muscle function tests, in particular the association between low muscle strength and the occurrence of complications [4]. Promoting physical capacity and fitness is essential in the current obesity treatment, but also before and after bariatric surgery. The goal of preoperative and postoperative physical therapy is to avoid distinctive muscle atrophy, which is an essential part of the postoperative care [3]. Also, as pointed out by the study of Gumieiro et al. [5], lower handgrip strength is associated with a vitamin D deficiency. In our Obesity Center, shuttle walks run test (SWRT) and handgrip strength (HS) measurement is included in the perioperative care of bariatric surgical patients. Vitamin D is pivotal for a good musculoskeletal and bone health [6]. Vitamin D deficiencies are often asymptomatic. In case of muscle weakness there is often a vitamin D deficiency and vice versa [6]. There is emerging consensus that serum vitamin D levels from ≥75–80 nmol/L are optimal for both bone health and skeletal benefits [7]. However, there are no procedure specific guidelines on how to achieve this target in patients following bariatric surgery. Maniscalo et al. [8] assessed the magnitude of difference in walking capacity and perceived symptoms in obese subjects after bariatric surgery. An improvement of distance walked in six min was seen one year after surgery. However, the effect of vitamin D levels and protein intake on these results has not been included. Our standard supplementation regimen consists of 800 IU oral cholecalciferol and 1000mg calcium carbonate from three weeks postoperatively. Additional supplementation with liquid cholecalciferol 50,000 IU monthly was deployed since January 2016, because 25 (OH) cholecalciferol deficiencies exist frequently with the standard supplementation regimen. However, it is unclear whether this change in cholecalciferol supplementation regimen affected the outcomes of physical fitness of bariatric patients. The aim of this study is to assess the effect of different cholecalciferol supplementation regimes and protein intake on physical fitness, measured using the HS and the SWRT. We hypothesized:

- Low blood levels of 25 (OH) cholecalciferol affect muscle strength and physical fitness and will result in a lower HS and decreased SWRT distance.
- A high dose of cholecalciferol monthly in addition to a standard daily dose gives better results regarding HS and SWRT distance.
- Higher protein intake improves the results regarding HS and SWRT distance.

## 2. Materials and Methods

### 2.1. Patient Selections

For this retrospective study we used a cohort of 100 patients who had bariatric surgery in the period of November 2015 until January 2016. All patients underwent a sleeve gastrectomy or Roux-en-Y gastric bypass procedure, in our Obesity Center. Patients with kidney disease or gastrointestinal disorders suggestive for malabsorption were excluded from this study. Group A (n = 50) used oral cholecalciferol supplementation with 800 IU per day. Group B (n = 50) used oral cholecalciferol supplementation with 800 IU per day and 50,000 IU cholecalciferol monthly lifelong. Both groups were matched for age, gender, preoperative body mass index (BMI), current BMI and surgical procedure. This study was approved by the Institutional Review Board of the Catharina Hospital Eindhoven (research registry database number 4944) and adheres the principles laid down in declaration of Helsinki in 1964.

### 2.2. Blood Collection and Biochemical Measurement

Serum cholecalciferol levels were measured three months preoperatively and six months postoperatively. In addition, as a part of our treatment protocol, calcium and parathyroid hormone (PTH) levels were measured. Vitamin D deficiency was defined as a serum vitamin D level < 75 nmol/L. Vitamin D (25-hydroxy vitamin D) was determined in serum by an immunometric competition assay on Liason^®^ using Diasorin^®^ reagents. Reference values of calcium were between 2.10 and 2.55 mmol/L and for PTH between 1.6 and 6.9 pmol/L.

### 2.3. Correction of 25 (OH) Cholecalciferol Deficiencies Preoperatively

A 25 (OH) cholecalciferol deficiency was defined by blood serum levels < 75 nmol/L and were treated with cholecalciferol in all deficient patients. Dosage: 50,000 IU per week for the first 6 weeks, afterwards monthly up to the bariatric procedure.

### 2.4. Handgrip Strength (HS)

The maximum grip strength of the hand is a good indication of the muscle function [1,4]. The muscle strength was evaluated by measuring the HS with the JAMAR hydraulic hand dynamometer (Lafayette Instruments, Lafayette, IN, USA). Patients were asked to sit on a comfortable chair with form armrests on which. The elbow of the patient was flexed at 90° and they were asked to squeeze the force meter with their preferred hand for approximately 2 s. Two measurements per patient were done. After each measurement the pointer of the meter was turned to zero and the best value was record. The HS was measured preoperatively, 3 months and 6 months postoperatively. Table 1 gives an overview of HS meter references in kilogram-force (kgf) [9].

### 2.5. Shuttle Walk Run Test (SWRT)

The SWRT is a reliable and valid test by repeatedly measuring the submaximal exercise functional capacity [10,11]. The patient runs up and down between 2 lines in a range of 10 m. The walking pace is indicated by a beep of a sound system. The patient leaves the first line as soon as a signal sounds. Patient walks to the second line where the patient should have arrived at the following sound. Per minute, the walking speed is increased by shortening the time between beeps. The test ends when the patient is too late two times in a row, or the rate cannot be maintained anymore. The SWRT was done preoperatively, 3 months and 6 months postoperatively. For our bariatric patients, the SWRT conform the Bradley protocol was used [12]. Outcome measurements are shown in metabolic equivalent of time (MET). MET’s have been determined for a wide variety of activities and are specific to that particular physical activity [12]. Each MET stage has been related to a particular level, speed in km per hour and a distance in meters (Table 2).

### 2.6. Protein Intake

Recommended protein intake was calculated conform the AACE/ASMBS guidelines to 1.0 g per kilogram ideal body weight (body mass index of 22.5 kg/m²) to a minimum of 60 g per day [13]. A 24-h food intake registration was done 3 and 6 months postoperatively and protein intake was calculated by a nutritionist.

### 2.7. Statistical Analysis

Data were retrospectively collected, managed, and analyzed using SPSS version 20, for Windows (SPSS Inc., IBM Corporation, Armonk, NY, USA). Quantitative data are denoted as mean ± standard deviation. Categorical variables were presented as frequency with percentages and the chi-square was used to compare these data. Data distribution was determined by assessing the skewness and kurtosis. Depending on the data distribution, either parametric tests (independent *t*-test, or student’s *t*-test) or non-parametric tests (Mann Whitney-U) were used to analyze the results. To assess the effect of protein intake on postoperative outcomes of the SWRT and HS a multivariate analysis was used. *p* Values ≤ 0.05 were considered statistically significant.

## 3. Results

Table 3 shows the baseline characteristics of both patient groups. Calcium levels in group A three months preoperatively and 6 months postoperatively were 2.37 ± 0.08 mmol/L and 2.39 ± 0.08 mmol/L (*p* = 0.058). Calcium levels in group B were respectively 2.38 ± 0.11 mmol/L and 2.38 ± 0.09 mmol/L (*p* = 0.930). The PTH levels in group A were respectively 7.5 ± 3.1 pmol/L and 6.5 ± 2.9 pmol/L (*p* = 0.032) and in group B 6.8 ± 2.7 pmol/L and 5.2 ± 1.6 pmol/L (*p* < 0.001). In group B there was a much more pronounced decrease in PTH level after 6 months (probably due to higher doses of cholecalciferol supplementation). Twenty-five (OH) cholecalciferol levels increased from 37.8 ± 20.6 to 66.7 ± 18.5 and from 47.0 ± 21 to 94.2 ± 25.7, for group A and B respectively (*p* = 0.001 for both groups). Of all the patients, 59 completed the HS preoperatively, 93 completed it three months postoperatively, and 99 patients completed the test 6 months postoperatively. Fifty-eight patients completed the SWRT preoperatively and eight-four patients at 6 months postoperatively.

### 3.1. Handgrip Strength

HS outcome measurements of group A and B pre- and post-operatively were shown in Table 4. No significant differences were found between group A and B postoperatively (*p* = 0.439). No significant differences in HS outcomes were found between patients with 25 (OH) cholecalciferol levels < 75 nmol/L or >75 nmol/L (Table 5).

### 3.2. Shuttle Walk Run Test

SWRT outcome measurements of group A and B pre- and post-operatively are shown in Table 4. No significant differences were found between group A and B postoperatively (*p* = 0.517). No significant differences in SWRT outcomes were found between patients with 25 (OH) cholecalciferol levels < 75 nmol/L or >75 nmol/L (Table 5).

### 3.3. Protein Intake

An adequate protein intake in relation to the calculated requirements was seen in 34 patients (68%) of group A and 41 patients (82%) of group B. The remaining 16 patients (32%) in group A and 9 patients (18%) in group B had a protein intake < 60 g/day. Using a multivariate analysis, the postoperative HS is significantly influenced by protein intake (*p* = 0.017). Protein intake has no significant influence of the postoperative outcomes of the SWRT (*p* = 0.447).

## 4. Discussion

This study aimed to assess the effects of two different cholecalciferol supplementation regimes of physical fitness measured with the HS and the SWRT before surgery, and three and 6 months after surgery. Outcomes of HS and SWRT tests were not significantly influenced by 25 (OH) cholecalciferol levels and it seems that protein intake plays a more important role in maintaining adequate muscle strength in this study. To our knowledge, this is the first study that assesses the influence of cholecalciferol supplementation in relation to physical fitness in bariatric surgical patients.

As pointed out by the study of Gumieiro et al. [5] lower handgrip strength is often present in patients with (or at risk of) a vitamin D deficiency. Vitamin D deficiency, when symptomatic is usually accompanied by muscle weakness and also muscle loss [6]. This indicated the important role of vitamin D in bone and musculoskeletal health. However, in our study no significant differences were found between patients with 25 (OH) cholecalciferol levels < 75 nmol/L and vitamin D levels >75 nmol/L.

On a histological level, myopathic changes were seen in muscle biopsy specimens of morbidly obese patents after two weeks of starvation [4]. Also, several reports on fat-free mass loss (FFML) in bariatric surgery showed that there is muscle loss after surgery [4,14]. These findings were substantiated by a review of Chaston et al. indicating that bariatric surgery results in greater FFML than very low-calorie diets [14]. The amount of %FFML is different among several bariatric surgical procedures. In the same review by Chaston et al. [14], the greatest %FFML was found in Biliopancreatic Diversion and Roux-en-Y Gastric Bypass procedures compared to adjustable Gastric Band. Unfortunately, no data was presented about the sleeve gastrectomy. The aforementioned findings might be an explanation for the significant decrease in handgrip strength in the postoperative period. The findings were corroborated by a recent study by Pouwels et al., 2015, that showed a significant decrease of respiratory muscle strength after bariatric surgery. However, it is difficult to assess one factor that is responsible for these changes. It seems that protein intake is more likable to be responsible for maintaining muscle strength after bariatric surgery and there is less effect of adequate cholecalciferol supplementation. However, the metabolic changes after bariatric surgery are mainly multifactorial. This was also shown in a study by Berggren et al., 2008, indicating that obese patients have an impaired beta-oxidation of lipids, that significantly improved with exercise training.

An inadequate protein intake will affect the results of physical fitness activities. The study of Stegen et al. [3] showed that a three times per week endurance and resistance exercise program could prevent this decrease and even induce an increase in strength in most muscle groups in the first four months postoperatively. However, in the studies of Chaston et al. and Stegen et al. [3,14] protein intake was not included. The study of Davies et al. [15] describes that protein loss in gastric restrictive procedures is considerably lower than in malabsorptive procedures. However, protein malnutrition and a decrease in fatty:lean mass ratio of 4:1 in certain restrictive procedures has also been reported in this study. The incidence of protein malnutrition in all purely restrictive procedures is between 0–2% and in all malabsorptive procedures between 13.4–18% [15]. Sixteen patients (32%) of group A and nine patients (18%) of group B had an inadequate protein intake in relation to the calculated requirements. In our study, we have found that an inadequate protein intake is significantly associated with the HS in the postoperative period, but not in the outcomes of the SWRT. This suggests that in terms of muscle strength protein intake is obviously very important but in terms of physical capacity it might be less important.

### 4.1. Clinical Implications of Handgrip Strength (HS)

Since muscle function reacts earlier to nutritional deprivation as well as restoration than muscle mass, it is obviously very tempting to employ HS as target for detecting and monitoring changes in nutritional status [4]. HS is a simple, noninvasive marker of the muscle strength of the upper extremities, which is well suitable for clinical practice. This parameter is easy to measure, resulting in only minimal costs. Moreover, an increasing number of studies have shown the predictive value of HS with regard to mortality and morbidity in a variety of clinical conditions [1]. Improvement in muscle function is usually accompanied by improved functional status. Norman et al. [1] showed an improvement in HS in the intervention group of malnourished patients with benign gastrointestinal disease. The intervention group have had oral nutritional supplements for three months and results were significantly correlated to physical function and role physical [1]. Beattie et al. [16] examined oral nutritional supplements for 10 weeks in malnourished surgical patients. Postoperative HS reduction in intervention patients was less marked, with significantly improved values at 10 weeks when compared with controls [16]. Ha et al. [17] showed a significantly higher increase of HS in malnourished stroke patients with nutritional support for three months. Paton et al. [18] reported significant increase in fat free mass and hand grip strength in malnourished tuberculosis patients after six-week intervention with sip feeds. All these results confirmed the necessity for a good nutritional status and adequate protein intake. In comparison to the other studies, the study of Otto et al. [1] showed no significant changes in HS during the first 4 months after bariatric surgery. Nevertheless, the preoperative HS showed a strong positive correlation with the postoperative body composition. However, nutritional status with a protein intake was not included in this study [1]. Besides that, age and gender are the strongest influencing factors on HS in healthy people [4]. However, the influence of gender and age has never been investigated in the bariatric target group. In our study, the study population is too small to make a good sub analysis in both groups.

### 4.2. Clinical Implications of the Shuttle Walk Run Test (SWRT)

The outcomes of SWRT in both groups were significantly increased after three and six months. No significant differences between both groups (standard and additional supplementation) were found which suggests that different cholecalciferol supplementation regimen seem to have a little impact on the outcomes. Multiple studies have shown that the SWRT is a reliable indicator for the assessment of cardiopulmonary fitness [19,20,21,22]. The study of Goncalves et al. [20] showed that female gender, older age and lower heart rate before the test are the determinants of not reaching maximal effort. In the current literature the SWRT and other measurement properties are widely used to determine the exercise capacity in a variety of diseases [10,11,12], however, there is no consensus which one is the (possible) gold standard. In current bariatric practice exercise capacity is measured by either objective measurements such as exercise bouts [23] and also by questionnaires [24]. In future research we need to assess possible differences in properties to measure exercise capacity and also its correlates with clinically relevant outcome measurements (e.g., vitamin status and protein intake).

### 4.3. Limitations

Despite the promising findings in this study, we also need to discuss limitations. Firstly, this was purely a retrospective study, which can give bias despite adequate matching of both groups. Secondly to really study the effects on muscle strength we also need to take into account other vitamins and minerals that play a pivotal role in musculoskeletal health. Thirdly effects on clinical outcomes need to be studied in a larger and better designed randomised study including DEXA scans. Due to small numbers in this study, the differences between Sleeve Gastrectomy and Roux-en Y Gastric Bypass were not studied.

## 5. Conclusions

We have found that different cholecalciferol supplementation regimes do not have a significant effect on physical fitness measured with the HS and SWRT before and three and 6 months after surgery. Both of them were not significantly influenced by low or normal 25 (OH) cholecalciferol. It seems that protein intake plays a more important role in maintaining adequate muscle strength.

## Figures and Tables

**Table 1:** Grip strength meter references in kilogram-force (kgf).

Age	Female	Male
15	28	42
20	29	43
25	30	44
30–45	30	45
50	29	45
55	28	44
60	27	43
65	25	41

**Table 2:** Metabolic equivalent references of a modified shuttle walk test [12].

MET Score	Level	Speed in km/Hour	Distance (Meter)
2.5	Level 3	3.0	80–120
3.0	Level 4	3.6	130–180
3.5	Level 5	4.2	190–250
4.0	Level 6	4.8	260–330
5.0	Level 7	5.4	340–420
5.5	Level 8	6.0	430–520
6.0	Level 9	6.6	530–630
6.5	Level 10	7.2	640–750
7.0	Level 11	7.8	760–880
8.0	Level 12	8.4	890–1020
9.0	Level 13	9.0	1030–1170

**Abbreviations:** MET = Metabolic Equivalent of Time.

**Table 3:** Baseline characteristics (n = 100) (mean ± SD).

	Group A	Group B	*P* Value
**Age (years)**	43.8 ± 11.6	47.5 ± 9.7	*p* = 0.075
**Gender (n)** **- Male:female**	9:41	10:40	*p* = 0.799
**Preoperative body mass index (kg/m²)**	42.6 ± 5.7	42.5 ± 5.2	*p* = 0.911
**Current body mass index (kg/m²)**	31.8 ± 4.6	31.7 ± 4.6	*p* = 0.879
**Procedures (n)** **- SG** **- RYGB** **- Revision surgery**	28 18 4	29 20 1	*p* = 0.359

Abbreviations: SG = Sleeve gastrectomy, RYGB = Roux-en-Y Gastric Bypass, SD = standard deviation.

**Table 4:** Comparing handgrip strength (HS) and shuttle walk run test (SWRT) outcomes of group A and B preoperatively, 3 and 6 months postoperatively.

HS	Preoperatively	3 Months Postop	6 Months Postop	*p* Value
**HS group A**	33.7 ± 12.2	32.2 ± 9.3	32.2 ± 8.0	*
**HS Group B**	36.3±9.8	34.1 ± 10.9	33.8 ± 10.2	**
**SWRT group A**	5.2 ± 1.1	6.1 ± 1.5	6.7 ± 1.5	***
**SWRT group B**	5.2 ± 1.1	6.0 ± 1.6	6.7 ± 1.5	****

Abbreviations: HS = handgrip strength, SWRT = shuttle walk run test, SD = standard deviation, *: Preoperative HS compared to HS 3 months postoperative (*p* = 0.301), preoperative HS compared to 6 months postoperative (*p* = 0.052), HS 3 months postoperative compared to HS 6 months postoperative (*p* = 0.078), **: Preoperative HS compared to HS 3 months postoperative (*p* = 0.040), preoperative HS compared to 6 months postoperative (*p* = 0.058), HS 3 months postoperative compared to HS 6 months postoperative (*p* = 0.018), ***: Preoperative SWRT compared to SWRT 3 months postoperative (*p* = 0.06), preoperative SWRT compared to 6 months postoperative (*p* = 0.07), SWRT 3 months postoperative compared to SWRT 6 months postoperative (*p* < 0.001), ****: Preoperative SWRT compared to SWRT 3 months postoperative (*p* < 0.001), preoperative SWRT compared to 6 months postoperative (*p* < 0.01), SWRT 3 months postoperative compared to SWRT 6 months postoperative (*p* = 0.005).

**Table 5:** Differences in HS and outcomes of SWRT in patients with vitamin D levels < 75 nmol/L and patients with vitamin D levels > 75 nmol/L (mean ± SD).

HS	Vit D < 75 nmol/L	Vit D > 75 nmol/L	*p* Value
**Preoperatively** Group A Group B	34.2 ± 12.7 36.4 ± 10.4	29.5 ± 6.4 35.2 ± 4.4	***p* = 0.842** ***p* = 0.874**
**6 months postoperatively** Group A Group B	32.4 ± 9.03 7.3 ± 7.6	31.8 ± 5.3 32.8 ± 10.7	***p* = 0.799** ***p* = 0.110**
**SWRT**	**Vit D < 75 nmol/L**	**Vit D > 75 nmol/L**	***P* Value**
**Preoperatively** Group A Group B	5.1 ± 1.4 5.2 ± 1.3	5.0 ± 0.0 5.8 ± 1.5	***p* = 0.842** ***p* = 0.474**
**6 months postoperatively** Group A Group B	6.9 ± 1.6 6.6 ± 1.3	6.2 ± 1.1 6.4 ± 1.6	***p* = 0.096** ***p* = 0.923**

Abbreviations: HS = handgrip strength, SWRT = shuttle walk run test, SD = standard deviation.
